# Optimizing the evidence for linkage by permuting marker order

**DOI:** 10.1186/1471-2156-6-S1-S61

**Published:** 2005-12-30

**Authors:** Gyungah Jun, Yeunjoo Song, Sudha K Iyengar, Robert C Elston

**Affiliations:** 1 Department of Epidemiology and Biostatistics, Wolstein Research Building R1300, Case Western Reserve University, 2103 Cornell Road, Cleveland, OH 44106, USA

## Abstract

We developed a new marker-reordering algorithm to find the best order of fine-mapping markers for multipoint linkage analysis. The algorithm searches for the best order of fine-mapping markers such that the sum of the squared differences in identity-by-descent distribution between neighboring markers is minimized. To test this algorithm, we examined its effect on the evidence for linkage in the simulated and the Collaborative Studies on Genetics of Alcoholism (COGA) data. We found enhanced evidence for linkage with the reordered map at the true location in the simulated data (*p*-value decreased from 1.16 × 10^-9 ^to 9.70 × 10^-10^). Analysis of the White population from the COGA data with the reordered map for alcohol dependence led to a significant change of the linkage signal (*p *= 0.0365 decreased to *p *= 0.0039) on chromosome 1 between marker D1S1592 and D1S1598. Our results suggest that reordering fine-mapping markers in candidate regions when the genetic map is uncertain can be a critical step when considering a dense map.

## Background

Errors in map order may originate from the use of general genetic maps, e.g., Marshfield, that are based on a limited number of meioses and can lead to incorrect marker order and poor estimates of interpolated recombination fractions [1]. Moreover, genetic map distances are dependent on rates of recombination that are known to vary across the genome [2]. Recently, single-nucleotide polymorphism (SNP) markers have drawn a great deal of attention, because of their denser coverage and reduced genotyping costs. However, the use of SNP markers in the context of traditional multipoint linkage analysis raises a concern, since the construction of a genetic map for the SNP markers is mostly conducted through sequence-based physical maps. Assembly or in silico mapping errors in marker order are not only plausible, but also occur more frequently than is widely publicized [3].

The subject of map order in linkage analysis has been investigated and several statistical approaches have been suggested: 1) include order errors as nuisance parameters through the use of profile likelihoods [4], 2) use three weighted multipoint LOD score statistics that incorporate information from all possible marker orders [5], and 3) utilize a novel scoring criterion that combines information from genetic and sequence-based physical maps [6]. Numerous algorithms, e.g., branch-and-bound [7], simulated annealing [8], and evolutionary strategy [9], have also been applied to determine the most accurate map order. However, the effect of map order on the identity-by-descent (IBD) distribution and its impact on linkage analysis has not been fully examined. With uncertainty in map order, it is difficult to justify the final map order with high confidence, if small changes of marker order between neighboring markers have a substantial impact on the IBD distribution between sib pairs, and hence affect the strength and shape of the linkage signal. We investigated the effect of map order in multipoint linkage analysis and developed a new marker-reordering algorithm based on the distribution over all sib pairs of allele sharing between neighboring markers. We tested our algorithm on both the simulated and Collaborative Study on the Genetics of Alcoholism (COGA) data from the Genetic Analysis Workshop (GAW) 14 data. In this report, we describe our algorithm and provide results.

## Methods

### Simulated data

We screened all the groups from the simulated data with a binary trait (b) to obtain datasets representative of moderate to strong linkage. We selected the Danacaa population from the simulated data; the sample was ascertained as nuclear families. To identify replicates that provided the most information, we searched all the replicates in the Danacaa population using LODPAL (S.A.G.E. [10]) with only the microsatellite markers, to identify replicates with the best and worst LOD scores. We selected REP001 (*N *= 700 in 100 pedigrees with 1,214 sib pairs) and REP085 (*N *= 693 in 100 pedigrees with 1,181 sib pairs) with the highest and lowest LOD scores, 5.97 and 1.86, respectively. Our goal was to determine whether we could improve the evidence for linkage either or both of the replicates after reordering the markers.

### Collaborative Studies on Genetics of Alcoholism (COGA) data

For the COGA data, we restricted the analysis to individuals of White descent (*N *= 1,219 in 115 pedigrees with 1,374 sib pairs). Prior to linkage analysis, we split two loops in the selected pedigrees. Two different measures of alcohol dependence were averaged to obtain the final measure of alcohol dependence, a semi-quantitative trait, and we adjusted for pack years and the interaction between sex and age at interview. We calculated values at age 80 and added them to the residuals from the final regression model. To obtain an appropriate Box-Cox transformation parameter, we used the SEGREG program in S.A.G.E. [10]. We found that the most parsimonious model was the two-mean recessive model, and the estimate of the transformation (power) parameter was λ = 1.294.

### Genetic map and markers

The genetic maps were given separately for microsatellite and SNP markers by the GAW organizers. For the simulated data, because the distances are given as recombination fractions (θ), we combined the SNP map with the microsatellite map using the Kosambi map function. For the COGA data the distances are given as Kosambi centimorgans for both SNPs and microsatellites, and for these data we simply placed the markers at the appropriate locations, interleaving SNPs with microsatellites.

Initially, we selected microsatellite markers with an average intermarker distance of 7 cM as the framework map to identify candidate regions for further pursuit. At locations where we obtained evidence for linkage, we increased the density of markers using SNPs to cover an average spacing of 3 cM both in the simulated and in the COGA data (Affymetrix).

In the simulated data, with prior knowledge of the true location, we selected the candidate region between markers D01S0021 and D01S0026, adding 11 SNP markers (C01R0047–C01R0057). We also selected regions with no evidence for linkage between marker D01S0016 and D01S0019, adding 7 SNP markers (C01R0035–C01R0041). For the COGA data, we selected the candidate region between marker D1S548 (0 cM) and D1S1631 (135.76 cM), based on our preliminary analysis and a previously published result [11]. Then, we investigated the order of the fine-mapping markers (SNP markers).

### Reordering Procedure

We used, for two neighboring marker loci, the estimated IBD distribution for each sibpair, i.e., the probabilities that the sib pair shares 0, 1, or 2 alleles IBD. Denoting these probabilities *f*_0_, *f*_1_, and *f*_2 _at one locus and , , and  at the other, our criterion to find the best order was to minimize the sum over all sibs and all pairs of neighboring loci, .

Our reordering algorithm comprises three basic steps that are applied iteratively: initializing a list of markers as framework markers, multipoint IBD calculation, and reordering. First, all MS markers were placed in the list of framework markers. Second, for a fine-mapping marker between two framework markers, the sum of Δ over all sibs was calculated between each pair of these three markers (multipoint IBD calculation) and the order of the three markers chosen that minimizes these sums. Third, the list of framework markers was updated to include the new fine mapping marker. The algorithm sequentially inserts fine-mapping markers among the framework markers until there are no more fine-mapping markers left. The GENIBD program and SIBPAL in S.A.G.E. [10] were used to obtain the multipoint IBD distributions, and to detect linkage, respectively.

## Results

**Figure 1:**
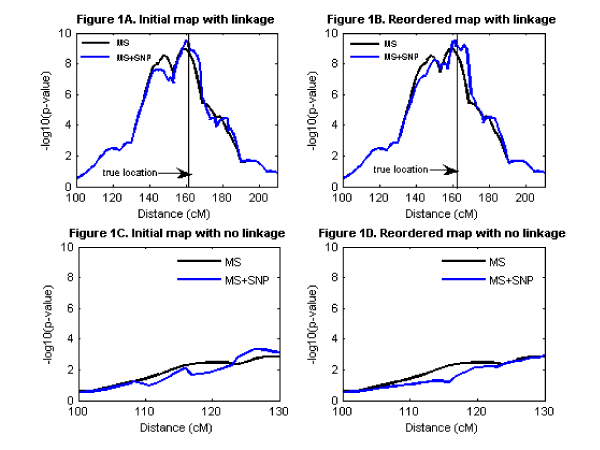
**Linkage analysis of the simulated data in REP001**. Linkage analysis of the simulated data using microsatellites only and microsatellites plus SNPs placed in order of the initial map (A and C) and the reordered map (B and D).

Using the initial map, the *p*-value for linkage at the true location in REP001 was 1.16 × 10^-9 ^(Figure 1A); after reordering the markers, the *p*-value was slightly decreased to 9.70 × 10^-10 ^(Figure 1B). Similarly, the linkage signal with REP085 was decreased at the true location from 1.59 × 10^-5 ^to 4.76 × 10^-6^. We observed that the linkage signal at regions with no linkage was either the same or reduced slightly after reordering the markers in REP001 (Figure 1C and 1D); the pattern was similar in REP085 (data not shown).

**Figure 2:**
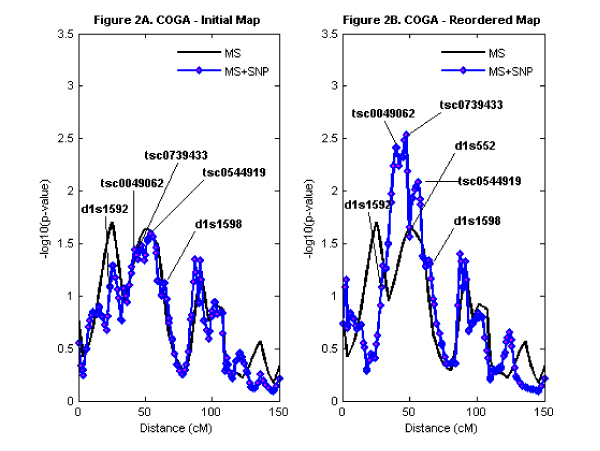
**Linkage analysis of the COGA data**. Linkage analysis of the COGA data using microsatellites only and microsatellites plus SNPs placed in order of the initial map (A) and the reordered map (B).

Increasing the map density by supplementing with additional SNP markers (Figure 2A) in the initial map (smallest *p*-value is 0.0231) did not show any noticeable change compared with using microsatellites only (smallest *p*-value is 0.0196). Using the reordered map (Figure 2B), the highest peak was found at tsc0739433 (SNP marker, 47.29 cM; *p *= 0.0029) compared to the initial estimate (49.47 cM; *p *= 0.0458). Table 1 lists the changes to the marker order in this region.

## Discussion

Previous analysis of the COGA data [11] also showed evidence of linkage on chromosome 1 using two-point linkage analysis when counting as unaffected those individuals who drink but have no symptoms of alcohol dependence. However, the signal was diminished using only data from White subjects. This may be because of the small number of sib pairs (~40) in the original dataset, compared with the current dataset (1,374 sib pairs).

For the simulated data, we simply combined the microsatellite and SNP maps with the Kosambi map function, which introduced uncertainty in the local map order between microsatellite and SNP markers. Hence, the small local shifts modified the multipoint IBD distribution sufficiently to be detected as changes in the linkage signal. Moreover, the COGA data showed there are considerable map uncertainties present in the initial map, and the average IBD calculation greatly affected by the local changes of marker orders.

In our study, we only considered marker order as the important parameter in construction of a genetic linkage map. However, a recent study [12] showed that several parameters, such as the number of meioses, intermarker distances, and marker heterozygosity, are also important to build more accurate genetic maps. In addition to these parameters, linkage disequilibrium should not be neglected, since ignoring linkage disequilibrium among tightly linked markers induces bias in the multipoint IBD distribution [13]. Finally, methods to estimate or impute the multipoint IBD sharing will also affect the methodology used by our group. Hence, these parameters along with marker order need to be carefully investigated in multipoint linkage analysis.

## Conclusion

We investigated the impact of map order on the IBD distribution, and developed a maker-reordering algorithm to optimize the linkage evidence. In both the simulated and the COGA data, we found an improvement in the linkage signal with the reordered map using our algorithm. We believe that a more generalized approach including additional parameters (e.g. linkage disequilibrium and marker informativity) incorporated with our algorithm will help to construct a more accurate genetic map, and consequently improve the process of multipoint linkage analysis.

## Abbreviations

COGA: Collaborative Studies on Genetics of Alcoholism

GAW: Genetic Analysis Workshop

IBD: Identity by descent

SNP: Single-nucleotide polymorphism

## Authors' contributions

GJ performed the overall statistical analysis and interpreted the data. YS prepared and analyzed the data. SKI designed the study, interpreted the results, and helped to draft the manuscript. RCE developed the algorithm, reviewed results, and helped to draft the manuscript.

## Figures and Tables

**Table 1:** Marker order between D1S548 (0 cM) to D1S1598 (61.8 cM) using the 3-cM map

Order	Microsatellites (7 cM)	SNP (3 cM)	Initial Map	Reordered Map
1	0	0	**D1S548**	tsc0273278
2	-	2.52	tsc0273278	**D1S548**
3	3.5	3.5	**D1S1612**	tsc0609730
4	-	5.78	tsc0609730	tsc0574502
5	-	8.56	tsc0574502	tsc0272985
6	-	10.69	tsc0272985	tsc0043572
7	-	14.09	tsc0043572	tsc0041639
8	-	15.72	tsc0041639	tsc1262794
9	-	18.28	tsc1262794	tsc0057485
10	-	21.12	tsc0057485	**D1S1612**
11	-	23.97	tsc0015353	tsc0015353
12	25.36	25.36	**D1S1592**	tsc1483792
13	-	29.05	tsc1483792	**D1S1592**
14	-	32.09	tsc0613868	tsc0613868
15	34.16	34.16	**D1S552**	tsc0617899
16	-	36.57	tsc0617899	tsc0052103
17	-	39.38	tsc0052103	tsc0049062
18	-	42.01	tsc0049062	tsc0390730
19	-	44.67	tsc0390730	tsc1030627
20	-	47.29	tsc1030627	tsc0739433
21	-	49.47	tsc0739433	**D1S552**
22	-	51.54	tsc0544919	tsc0502862
23	-	54.29	tsc0502862	tsc0544919
24	-	57.90	tsc0054968	tsc0054968
25	59.09	59.09	**D1S1598**	tsc0061295
26	-	61.85	tsc0061295	**D1S1598**
27	-	64.90	tsc0914793	tsc0914793

Bold letters are microsatellite (framework) markers.
